# Integrating Attenuated Total Reflectance–Fourier Transform Infrared Spectroscopy and Multidetector Computed Tomography for Analysis of Heat-Induced Changes in Bone

**DOI:** 10.3390/ma18040742

**Published:** 2025-02-07

**Authors:** Tamara Leskovar, Fabio Cavalli, Lea Legan, Dario Innocenti, Polonca Ropret, Matija Črešnar

**Affiliations:** 1 Centre for Interdisciplinary Research in Archaeology, Department of Archaeology, Faculty of Arts, University of Ljubljana, Zavetiška 5, 1000 Ljubljana, Slovenia; tamara.leskovar@ff.uni-lj.si; 2 Research Unit of Paleoradiology and Allied Sciences, TS—SCIT, Azienda Sanitaria Universitaria Giuliana Isontina, Via della Pietà, 2/1, 34100 Trieste, Italy; fabio.cavalli@asugi.sanita.fvg.it (F.C.); dario.inox@gmail.com (D.I.); 3 Institute for the Protection of Cultural Heritage of Slovenia, Poljanska Cesta 40, 1000 Ljubljana, Slovenia; lea.legan@zvkds.si (L.L.); polona.ropret@zvkds.si (P.R.); 4 National Museum of Slovenia, Prešernova Cesta 20, 1000 Ljubljana, Slovenia; 5 Smithsonian Museum Conservation Institute, 4210 Silver Hill Road, Suitland, MD 20746, USA; 6 Faculty of Chemistry and Chemical Technology, University of Ljubljana, Večna pot 113, 1000 Ljubljana, Slovenia

**Keywords:** skeletal remains, ATR-FTIR spectroscopy, Multidetector CT, FTIR indices, density, cremation

## Abstract

Charred and burned human and animal skeletal remains are frequently found in archaeological records and can also be subjects of forensic investigations. Heat exposure causes specific changes to the physical and chemical characteristics of these remains, offering valuable insights into their taphonomic history. This research combines the commonly used ATR-FTIR (Attenuated Total Reflectance–Fourier Transform Infrared) spectroscopy with the rarely utilised density measurements obtained from Multidetector CT (Computed Tomography) to investigate changes in defleshed bovine cortical bone exposed to different temperatures for varying durations. The inclusion of density measurements is significant because Multidetector CT analysis is non-destructive and can be valuable when remains cannot be removed from their burial context (e.g., urn) or cannot be damaged. The results indicate complex changes in both organic and inorganic components, affecting crystallinity and density. Lower temperatures primarily affect organic matter, while higher temperatures induce significant changes in the mineral lattice and phase transitions. The transformation from β-tricalcium phosphate to α-tricalcium phosphate at high temperatures likely impacts the bone’s crystallinity and density. Bone density measured by CT scans provided additional information that complemented the interpretations of FTIR spectroscopy. While CT scans offer important data for planning non-destructive analyses of remains, they present only one layer of information. Therefore, CT scans need to be combined with other techniques to provide comprehensive interpretations of the changes occurring in the bone. Further research is needed on density measurements and other potentially non-destructive analyses to fully unlock the potential of Multidetector CT analyses.

## 1. Introduction

Be it the result of cooking practices, mortuary and other rituals, destruction of evidence or anything in between, charred and burned human and animal skeletal remains are found in many archaeological and forensic contexts. As exposure to heat leaves a unique pattern in the structural and chemical characteristics of remains, they present a powerful source of understanding regarding the taphonomic history of the remains. In archaeological contexts, they can provide a rich source of information regarding life in the past, while in forensic contexts, they provide means for the reconstruction of events and possible identifications [1,2,3].

However, bones are a composite material, which makes understanding the effect of heat on their structural and chemical characteristics far from straightforward. Bones can be divided into cancellous and compact types, composed of water (approximately 10%) and closely linked organic (approximately 25%) and inorganic phases (approximately 60–70%). The former is mainly represented by lipids and proteins, 90% of which are type I collagen, formed by amino acid chains in a triple-helical structure [4,5]. Collagen molecules in the bone form elastic fibrils, held together by hydrogen bonds [6]. In the empty spaces between collagen molecules and fibrils are inorganic plate-shaped nanocrystals of non-stoichiometric (highly disordered) apatite with predominantly carbonate (3–8%) but also other (e.g., Na, Mg, Al, Pb, HPO_4_) substitutions replacing phosphates and hydroxy groups [5,7,8,9,10,11,12,13]. Furthermore, bone mineral is hydrated as it contains water in its structure [14].

With exposure to heat, processes such as combustion and pyrolysis (thermolysis) change the structural and chemical characteristics of bones. To simplify, the changes that occur can be summarised as a loss of water (<250 °C), followed by a decline in organic components (200–600 °C) and changes in the mineral (predominantly > 700 °C). Most free water is lost as the temperature approaches 100 °C and structural water is lost as the temperature approaches 250 °C, with some small, additional changes around 400 °C, when water produced as a byproduct of organic matter combustion evaporates. Lipids completely degrade at temperatures of around 300–340 °C. Between 200 and 350 °C, a loss of collagen is observed. The majority of the organic phase is combusted between 300 and 400 °C in oxidising conditions and 600 and 700 °C in reducing conditions. Collagen is converted into alkylated benzenes and phenols and condensed aromatic compounds, which decline from around 500 °C onwards and almost disappear by 900 °C. At temperatures above 600–700 °C, cyanamide forms due to a reaction between the residual C and N from organic matter and calcium from the inorganic part of the bone [15,16,17,18].

Some minor changes in the mineral part of the bone can be observed between 250 and 340 °C, likely correlated with the changes in organic content. Most of the changes in bioapatite occur at temperatures above 600–700 °C, although an increase in crystal size, which is non-linearly correlated with increasing temperature, can start between 300 °C and 500 °C. Due to the increase in crystal size, at temperatures above 500 °C, a slow increase in density occurs, which becomes faster at temperatures above 700 °C. Most alterations in the bone mineral can be seen between 800 °C and 900 °C, when growth of the crystals and ordering of the crystal structure, accompanied by the loss of carbonates and structural water, are most pronounced. Structural carbonate loss starts at around 250–340 °C; however, most of the loss occurs at temperatures above 600 °C. Between 700 °C and 900 °C, in oxidising conditions, an increase in hydroxyl ions can be observed, when carbonate ions reorganise into carbon trioxide to create space for them. Additional carbonate losses occur at temperatures between 700 °C and 1000 °C, with some minor losses reported at temperatures up to 1100 °C [16,17,18].

Previous studies investigating bone hardness, porosity and density after exposure to high temperatures presented decreasing hardness with exposure to temperatures between 100 °C and 300 °C. The lowest hardness was observed to be between 400 °C and 500 °C, which remained low until exposure to temperatures of 800–900 °C, when it started to rise again [19,20,21,22], supposedly due to the increasing density of hydroxyapatite and crystal growth [23,24,25]. Bone porosity was highest in the samples exposed to temperatures of 600 °C (around 50%), while with increasing temperatures, porosity decreased, with sintering occurring at approximately 1200 °C [26].

Analysing these changes in bones can offer information regarding the reasons and methods for exposing remains to heat. Thus, there are numerous studies focusing on heat-induced changes [27,28,29,30,31,32,33,34,35]. Although there are various techniques used for this purpose, vibrational spectroscopy, especially Fourier Transform Infrared (FTIR) spectroscopy, is one of the most common choices [28,31,32,34,35,36,37,38,39,40,41,42,43,44,45]. This is likely because FTIR spectroscopy is a convenient and relatively inexpensive technique for the exploration of molecular structure and material characterisation. Additionally, the Attenuated Total Reflectance (ATR) technique is increasingly being used as it minimises sample preparation, improves signal-to-noise ratios and increases comparability among samples [46,47]. When used on skeletal tissues, FTIR spectroscopy allows researchers to explore the organic (mainly collagen) and inorganic (bioapatite) parts of the tissues. Due to its precision and well-established use in research on skeletal tissues, it also enables detection of small chemical and/or structural variations in the samples [31,33,48,49]. However, the chemical properties of skeletal tissues are heterogeneous, making molecular characterisation using FTIR spectra elusive [31]. To characterise the molecular structure of skeletal samples, calculations of the ratios between peak heights and/or areas are necessary, ensuring that the results present relative and not absolute concentrations of components in the sample [50].

Another less-known technique for studying skeletal tissues is Multidetector CT, which enables the acquisition of three-dimensional (3d) volume images. It is gaining popularity in archaeological research, especially for analysing ancient cinerary, as it can reveal the contents of urns [51,52]. A wide X-ray beam is used in conjunction with multiple rows of detectors to acquire more than one CT slice in a single tube rotation and to simultaneously avoid tube overloading. In general, a Multidetector CT slice consists of a matrix of the smallest elements called “voxels”. The coefficient of absorption at each voxel is converted into a CT value, reported in Hounsfield units (HUs), which is defined by Equation (1) as follows:

(1) $$\mathrm{CT}\;\mathrm{value}=k\times \frac{\mu_{t}-\mu_{w}}{\mu_{w}}$$

where µ_t_ is the coefficient of absorption at the scanning volume, µ_w_ is the coefficient of absorption with respect to water and k is a proportionality constant, which is fixed at a value of 1000.

Hounsfield unit (HU) values are defined as a measurement of the linear attenuation coefficient of a tissue, based on a defined scale of 0 for the radiodensity of distilled water at standard pressure and temperature and −1000 for air at standard pressure and temperature. In CT scans, the reconstruction software calculates HU values for each volume unit (voxel) of the scan [53].

The density of cortical bone varies depending on whether it is measured in living bone or in bone remains, including historical/archaeological ones. However, ranges vary between 1150 and 1550 HU. In skeletal remains, there is some loss of the mineral component of the bone due to taphonomic processes. Cremated bones instead seem to have a higher density, generally over 2000 HU and up to more than 2500 HU, probably due to changes in the composition of the mineral component and alterations in the morphology and size of crystallites of bone hydroxyapatite [54]. Preliminary experiments seem to express a certain correlation between the density of the cortex of the cremated bone and the cremation temperature [55].

### Aims of the Research

The vast amount of research using FTIR spectroscopy to address changes in bones exposed to heat has allowed researchers to establish FTIR indices with relatively well-known meanings (see Table 1). However, there are still some gaps in our understanding of FTIR results and heat-induced changes, one being the effect of time of exposure. Although it was previously observed that time of exposure does play a role [40,56], it is not commonly and systematically included in research designs. Thus, the first aim of this study was to address the influence of time of exposure on changes in bones observed with FTIR spectroscopy.

Contrarily to FTIR spectroscopy, bone density calculated from CT scans is not commonly used to understand heat-induced changes in bones. As it could provide a completely non-destructive technique for assessing information about bones, this study expands the commonly applied FTIR indices with CT density. First, a set of baseline changes was established using calculated indices from the FTIR spectra. In the next step, density calculations were performed and compared against the FTIR indices. The second aim of this study was to establish a correlation between FTIR indices and density to better understand what information density can tell us about the bone. Since Multidetector CT is a non-destructive method, which can be performed without disturbing the burial context, the aim was to determine if it could be used as a standalone technique for studying and interpreting taphonomic changes in remains or if other methods need to be incorporated.

Research was conducted on a single bone with known taphonomic history to eliminate the influence of individual variability and the unknown complexities of bone diagenesis. This approach was chosen to isolate the effects of time and temperature of exposure, as FTIR spectra and density are known to be significantly influenced by extrinsic factors (e.g., post-depositional environment) and intrinsic factors (e.g., age, sex, skeletal element) [30,33,57,58,59]. By focusing on a single specimen, the study effectively controlled for these variables, ensuring that the observed changes were directly attributable to the experimental conditions. This method provides a clearer understanding of how time and temperature impact bone properties, free from the confounding effects of natural variability.

**Table 1:** Included FTIR indices.

Index	~Peaks	Characterisation	Reference
IRSF	(560 + 600)/585	crystallinity, indicating crystal size and order in the matrix	[60,61]
H1640	1640 height	relative amount of Amide I	[62,63]
H1415	1415 height	relative amount of type B carbonates + organic matter	[28,64,65]
OH	630 height	relative amount of hydroxyl groups	[32]
CN	2015 height	relative amount of cyanamide	[32,66]
H960	960 * height	relative amount of well-crystallised apatite	[67]
H872	872 * height	relative amount of B-type carbonates	[68]
H878	878 * height	relative amount of A-type carbonates	[68]

* Peaks measured in second-derivative spectra.

## 2. Materials and Methods

### 2.1. Samples

The mid part of the femur diaphysis of a single adult bovine was manually defleshed and cut into rectangular samples of 4 × 4.5 cm. One sample was used as a control while sixteen others were exposed to low, mid and high temperatures in a vented muffle furnace. Each sample was placed into the furnace individually once the set temperature was reached. Samples were exposed to temperatures of 300 °C, 600 °C, 900 °C and 1200 °C for 30, 60, 90 and 120 min each. After exposure, samples were naturally cooled to room temperature and stored inside perforated polyethylene bags.

### 2.2. ATR-FTIR Spectroscopy

Each sample was analysed using a Perkin Elmer Spectrum 100 FTIR spectrophotometer in Attenuated Total Reflectance (ATR) mode, equipped with a thallium–bromoiodide (KRS-5) crystal. To ensure full physical contact between the samples and the ATR setup, firm and consistent pressure was applied during measurements to optimise the interaction between the sample surface and the ATR crystal. The samples were measured in their original form, with full pieces of bone used; they were not crushed or altered. ATR spectra were collected in the range between 4000 cm^−1^ and 380 cm^−1^. For each sample, measurements were taken at five distinct spots, with 32 scans collected at a spectral resolution of 4 cm^−1^ per spot. All spectra underwent ATR correction using Spectrum software (Perkin Elmer, Norwalk, CT, USA). Additionally, the spectra were baseline-corrected, normalised (Min-Max normalisation) to the highest peak at ~1010 cm^−1^ and averaged using the OPUS 7.0 software package (Bruker, Bremen, Germany).

As most of the peaks used to understand the changes in the bone due to heat exposure were found to be between 550 cm^−1^ and 2020 cm^−1^, scanned spectra were reduced to wavelengths between 480 cm^−1^ and 2100 cm^−1^. Due to overlapping, three peaks were measured in second-derivative spectra using the Savitzky–Golay filter with 7 smoothing points (Supplementary Materials Figures S1 and S2).

FTIR indices were measured/calculated (Supplementary Materials, Figures S1 and S2) to help better understand the molecular structure of the bones. Indices were chosen based on the published research studying bones exposed to high temperatures (Table 1). Although there are more indices mentioned in various other studies, only the ones where peaks were clearly measurable for all the samples were included. Due to the normalisation of the spectra to the PO_4_ peak at ~1010 cm^−1^, some of the peaks, namely OH and CN, were slightly adjusted and a peak height of ~1010 cm^−1^ was used for the ratio calculation instead of ~605 cm^−1^.

### 2.3. CT Scanning

After cremation, the samples were scanned with a 16-slice Multidetector CT scanner (Toshiba Aquilion 16, 129 KVp, 200 mA, isotropic 0.5 mm voxel). The HU value of each voxel of the sample was registered and classified by frequency. In order to exclude the contribution of the partial-volume effect due to the micro-cracking of the bone structure caused by the heat, the histogram was deconvoluted to obtain the mean HU value of the higher component of the curve (CT).

### 2.4. Statistical Analyses

Statistical analyses were performed using IBM SPSS Statistics. First, normality of the data was checked using the Shapiro–Wilk test. Based on the results, parametric tests for normally distributed and non-parametric tests for not-normally distributed data were conducted. Spearman correlation was used to observe correlations between different FTIR indices and the CT density index. A confidence interval of 95% was used for means or medians to explore the degree of change in the bones due to exposure to heat and to evaluate their significance.

## 3. Results

All the obtained FTIR indices and densities are presented in the Supplementary Materials Table S1 and averaged measured/calculated FTIR and CT density indices are presented in Table 2.

### 3.1. Changes with Time and Temperature

**Figure 1:**
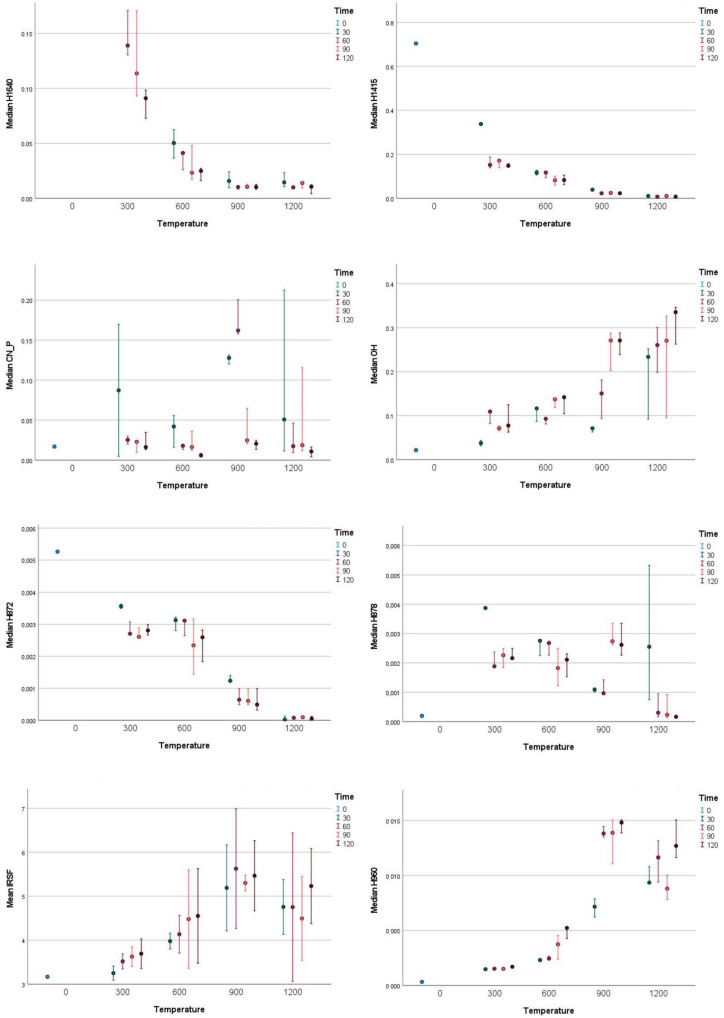
Confidence intervals of FTIR indices for different temperatures and durations of exposure.

The obtained FTIR results (Figure 1) showed that with exposure to temperatures of 300 °C, significant changes had already occurred in the bone. Significant decreases in H1640, H1415 and H872, as well as a significant increase in H878, had already occurred after 30 min of exposure. An increase in OH also occurred but was only clear after 60 min of exposure at 300 °C and there were no further significant changes until prolonged (90 min) exposure at 900 °C. IRSF slightly increased at 300 °C, continuing to gradually increase with prolonged exposure, as did stoichiometric phosphate (H960), but the latter presented no differences with prolonged exposure.

At 600 °C, H1640 continued to slowly decrease and was lost after 90–120 min of exposure. Similarly, H1415 continued to decrease; however, the changes were small and barely significant in the first 30 min of exposure and again after 90 min of exposure. H872 and H878 presented non-significant but observable decreases after at least 90 min of exposure, while at the same time, OH presented a similar pattern of increase. IRSF continued to increase slowly and gradually, as did H960; however, the latter presented a more pronounced increase upon 90 min and 120 min of exposure.

At 900 °C, H1640, H1415 and H872 were almost completely lost. In some cases, some remnants might still be detectable in the first 30 min. On the other hand, H878 and OH both initially decreased and then significantly increased after 90 min and 120 min of exposure. CN significantly increased after 30 min and 60 min of exposure and significantly decreased after 90 min and 120 min of exposure. The increase in IRSF was still present but stopped after 90 min and 120 min of exposure, while H960 showed an extreme increase after 60 min of exposure with no further significant change.

At 1200 °C, H1640, H1415, H872 and H878 has been largely removed. In some cases, some leftovers of H878 might still be visible after 30 min of exposure, and the same goes for CN, which seemed to persist for a bit longer, with peaks sometimes still detected after 60 min and 90 min of exposure. IRSF and H960 both experienced a decrease at 1200 °C exposure. IRSF decreased non-significantly, without any obvious differences caused by different time of exposure. H960 decreased significantly with some noticeable variation due to time of exposure, presenting significant differences between 30 min and 120 min of exposure, as it started to increase again with prolonged time of exposure. The OH peak seemed to resemble the peaks at 900 °C, although an increase was seen with exposure at 1200 °C for 120 min.

**Figure 2:**
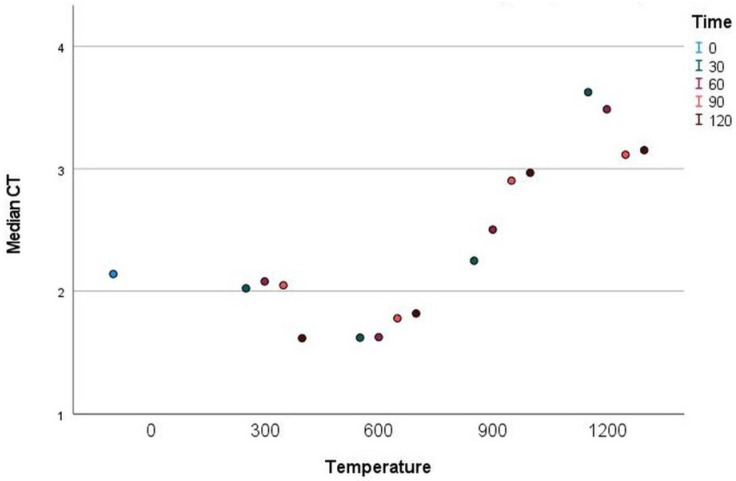
CT density for different temperatures and durations of exposure.

CT density only significantly changed after 120 min of exposure at 300 °C, when it decreased. The next significant change only occurred after 90–120 min of exposure at 600 °C; however, a more substantial increase was observed after exposure at 900 °C, which continued until 30 min of exposure at 1200 °C. With 60–120 min of exposure at 1200 °C, CT density decreased again (Figure 2).

### 3.2. Correlations

Considering the observed changes in CT density and FTIR spectroscopy measurements, the correlation analysis was adjusted as follows:

(1) First decrease in density (300 °C/120 min–600 °C/60 min): strong negative correlation with H1640 (0.84) and H1415 (−0.74); strong positive correlation with IRSF (0.84) and H960 (0.79).
(2) First small increase in density (600 °C/90–120 min): strong negative correlation with CN (−0.88); moderate positive correlation with H960 (0.68).
(3) First high increase in density (900 °C/30–60 min): strong negative correlation with H872 and H1415 (−0.88); strong positive correlation with H960, CN and OH (0.88).
(4) Second high increase in density (900 °C/90 min–1200 °C/30 min): strong negative correlation with H872 and H1415 (−0.80); moderate negative correlation with H960 and IRSF (−0.64).
(5) Final decrease in density (1200 °C/60–120 min): strong negative correlation with H1415 (−0.79); moderate negative correlation with H872 (−0.53); moderate positive correlation with H960 (0.53).

## 4. Discussion

### 4.1. FTIR Spectroscopy

Changes at 300 °C indicated loss of organic matter (H1640, partially H1415) following a short period (30 min) of exposure. Changes in the mineral lattice also took place in these early stages, as losses of B-type carbonates (H872) were accompanied by increases in A-type carbonates (H878), crystallinity (IRSF) and stoichiometric phosphates (H960). With prolonged exposure, further losses of collagen occurred (H1640), hydroxyl ions (OH) were incorporated into the lattice and crystallinity further increased (IRSF), while both types of carbonates (H1415, H872 and H878) and stoichiometric phosphates (H960) were not affected by the additional time of exposure. Significant changes in the organic part are to be expected [16,17,18], while the observed changes in the mineral part might predominantly be a consequence of changes in the organic part, as some of the spectral locations are shared and calculated ratios only present relative concentrations of specific molecules in the bone.

The presence of the Amide I peak (H1640) at 600 °C and its small, gradual decrease were visible due to the conversion of collagen into aromatic compounds [17]. Decreases in all type of carbonates (H1415, H872 and H878), especially with prolonged (90–120 min) exposure, indicated the non-selective loss of carbonates, which predominantly occurred only after prolonged exposure. On the other hand, a gradual increase in crystallinity (IRSF) was accompanied by an increase in stoichiometric phosphates (H960) and the incorporation of hydroxyl ions (OH) into the mineral lattice. These changes were consistent with reported reorganisation of the mineral lattice due to carbonate losses and crystal growth [16,17,31,69].

Although some significant changes in the mineral already occurred with prolonged exposure at 600 °C, they became more pronounced at higher temperatures. Furthermore, with prolonged exposure at 900 °C, a clear distinction between continuingly decreasing B-type carbonates (H872) and increasing A-type carbonates (H878) occurred, which was accompanied by an increase in hydroxyl ions (OH) and a decrease in cyanamides (CN). The latter presented a tangible increase during short periods of exposure (30 min and 60 min) and decreased significantly with prolonged (90 min and 120 min) exposure. The predominant loss of B-type carbonates observed agrees with the findings of previous studies [31,32,48,68], although one should take into account the very low relative amounts of A-type carbonates in the bone [70,71]. When acknowledging hydroxyl groups, cyanamides and A-type carbonates together, an interesting interplay can be seen. Short-term exposure resulted in an increase in cyanamides, accompanied by a decrease in A-type carbonates and hydroxyl groups, while with prolonged exposure, the opposite occurred: there was a decrease in cyanamides, accompanied by an increase in A-type carbonates and hydroxyl groups. When considering only samples exposed at 900 °C (hydroxyl peaks are known to increase only after exposure at 700 °C) [26,31], there was a strong negative correlation between A-type carbonates and cyanamides (r = −0.74), a moderate negative one between hydroxyl groups and cyanamides (r = −0.64) and a strong positive one between A-type carbonates and hydroxyl groups (r = 0.76). Thus, at 900 °C, cyanamides increased while A-type carbonates and OH decreased. Previous observations indicate that during heating, A-type carbonates are lost and replaced by hydroxyl groups [32]. When also considering cyanamides, Snoeck et al. [40] proposed that, when present, cyanamides substitute for hydroxyl groups and replace A-type carbonates. However, based on our results, at 900 °C, A-type carbonates and hydroxyl groups seemed to act together, with cyanamides on the other side. This only changed with complete loss of both A-type carbonates and cyanamides following prolonged exposure at 1200 °C, when only hydroxyl groups were left and were further incorporated into the lattice. The presence of cyanamides mostly correlated to the combustion of organics, namely to the heating of ammonia [72], in the case of bone possibly deriving from organic matter (collagen) [40]. Thus, it is surprising that they were still detectable at 1200 °C. As some leftovers of aromatic compounds were also present, they might be “feeding” the peak of cyanamides. A strong positive correlation was also seen between CN and H1640 (0.83) following short-term exposure at 1200 °C, almost completely disappearing (0.26) with prolonged time of exposure. With complete loss of cyanamides and A-type carbonates, only the hydroxyl group remained as substitutes in the lattice and were thus able to further increase. At this point, IRSF did not seem to change significantly, even decreasing slightly. This decrease had been previously noted in [31,32], but the reasons behind it are unclear, generally being attributed to the rearrangement of the apatite structure. Legan et al. [36] noticed additional bands characteristic of α-tricalcium phosphate (TCP) after exposure at 1200 °C, which agrees with a thermal transformation of β-TCP to α-TCP, occurring at approximately 1150 °C [73]. Since β-TCP has a rhombohedral, more stable structure with uniformly distributed Ca vacancies when compared to monoclinic α-TCP [74,75], transformation to α-TCP might be associated with the decreasing crystallinity.

### 4.2. CT-Obtained Density

The presented results showed an initial significant decrease in density with prolonged exposure at 300 °C, followed by a gradual increase until the period of prolonged exposure at 1200 °C. A decrease in density following exposure at 600 °C, followed by an increase upon exposure at 900 °C and 1200 °C, was also observed by Figueiredo et al. [26]. It was explained by changes in bone porosity caused by a loss of collagen at lower temperatures and complete removal of carbonate from the mineral lattice at higher temperatures. Based on the FTIR indices and correlations, our results partially agree with these interpretations. It seems like density only starts to change with prolonged exposure to low temperatures (300 °C) or after a short period of exposure to medium temperatures (600 °C). Correlations indicate that changes in the density occur due to losses of organic matter with the initial changes in crystallinity. However, according to FTIR indices, the latter are significantly less substantial, leading to lower density. This agrees with previous research showing that most of the water and organic matter is lost at temperatures between 300 °C and 800 °C when bone losses equate to approximately 30–45% of its weight [21,69,76,77,78]. Since the presence of organic matter protects inorganic components of the bone [69,78] and bone hydroxyapatite remains unchanged up to high temperatures [21,79], the results are understandable. Significant increases in density only occurred with exposure to high temperatures (900 °C), a phenomenon also observed by other researchers [26]. However, the reasons for the increase were more complex than just the loss of carbonates from the matrix. B-type carbonates were removed, but they were replaced, first by cyanamides and hydroxyl groups, and with prolonged exposure, following the removal of cyanamides, by A-type carbonates and hydroxyl groups. Although organic matter and B-type carbonates were removed, other molecules were being incorporated into the mineral matrix. Furthermore, at these temperatures, increases in stoichiometric apatite and crystallinity were observed, altogether likely causing density to increase. With longer exposure to very high temperatures (1200 °C), cyanamides and A-type carbonates were also removed and only hydroxyl groups persisted as substitutions. Although the mineral matrix was becoming “cleaner”, density and crystallinity both decreased. The latter is possibly due to the rearrangement of the apatite structure [31,32], while the decrease in density is unusual, if we assume crystal growth occurred and a closely interlocked structure was produced [69]. It might be due to the observed transition of β-TCP to α-TCP, as the former has higher density than α-TCP (3.07 g/cm^3^ vs. 2.86 g/cm^3^) [80,81,82].

## 5. Conclusions

This study aimed to improve our knowledge of heat-induced changes in bones observable with FTIR spectroscopy, also acknowledging the time of exposure and exposure to very high temperatures (1200 °C). Furthermore, it correlates relatively well-established FTIR indices with the simultaneous changes in bone density measured with CT.

The exposure of bone to varying temperatures results in complex changes in both organic and inorganic components, influencing crystallinity and density. Lower temperatures primarily affected organic matter, while higher temperatures induced significant mineral lattice changes and phase transitions. The transformation from β-TCP to α-TCP at high temperatures likely impacts the crystallinity and density of the bone. The changes at different temperatures can be summarised as follows:

**Low-Temperature Changes (300 °C):**
  - Initial exposure leads to a loss of organic matter and changes in the mineral lattice.
  - Losses of B-type carbonates coincided with increases in A-type carbonates, crystallinity and stoichiometric phosphates.
  - Prolonged exposure resulted in further collagen loss, the incorporation of hydroxyl ions into the lattice and increased crystallinity.

**Medium-Temperature Changes (600 °C):**
  - Collagen converted into aromatic compounds.
  - Crystallinity and stoichiometric phosphates increased, accompanied by hydroxyl ion incorporation.
  - There was a non-selective loss of all carbonate types with prolonged exposure.
  - There was an initial significant density decrease due to the loss of organic matter.

**High-Temperature Changes (900 °C):**
  - There was interplay between hydroxyl groups, cyanamides and A-type carbonates; short-term exposure resulted in an increase in cyanamides, accompanied by a decrease in A-type carbonates and hydroxyl groups, while with prolonged exposure, the opposite occurred.
  - There was a significant density increase due to interplay between hydroxyl groups, cyanamides and A-type carbonates, and an increase in stoichiometric apatite and crystallinity.

**Very-High-Temperature Changes (1200 °C):**
  - Complete loss of cyanamides and A-type carbonates.
  - Hydroxyl groups continued to increase.
  - Density and crystallinity decreased with prolonged exposure, possibly attributed to the transition from β-TCP to α-TCP.

The observed changes in the FTIR indices agreed with previous studies, while also offering a possible explanation—the transition from β-TCP to α-TCP—for the decreasing crystallinity previously observed with exposure to very high temperatures. Additionally, the study revealed significant differences between the short- and long-term exposure of the bones to different temperatures, indicating that time, although often neglected, is also a crucial factor in heat-induced changes. Because samples originated from a singular bone with known taphonomic history, the observed changes can directly be correlated to the exposure conditions. As such, the results provided a solid baseline for understanding the components of temperature and time, and the correlations between changes observed in FTIR indices and CT density. This is particularly important when working with valuable heritage materials. Firstly, FTIR analyses were performed on a piece of bone and not on powder, limiting the destructiveness of the method. Furthermore, Multidetector CT is a non-destructive technique. It can be, for instance, carried out without disturbing the burial context (e.g., urn) and/or damaging the remains (e.g., human bones). When results of Multidetector CT were analysed alongside FTIR indices, which offer analysis of several layers of data (changes in different molecules attributed to the organic and mineral phases of the bone), CT density only provided one value. Although there was a pattern of initial decrease, followed by a significant, gradual increase and another decrease in CT density, it would be hard to classify most of the samples in terms of the explicit (time and) temperature to which they were exposed. CT density could be a useful parameter to study bones exposed to heat but, for now, not as a standalone technique. Future research should include other parameters, also obtainable in a non-destructive way (e.g., porosity, microstructure), to interpret the taphonomic changes in bones exposed to heat.

The results offer meaningful insights and thus a sort of baseline for investigating the chemical and structural changes occurring in bones exposed to different temperatures and for different times. This could be useful when trying to decipher mortuary and other rituals, and also in forensic cases of combustion of the body. It was relatively easy to distinguish between samples exposed to different temperatures and, to an extent, times, acknowledging all the FTIR indices and density measurements using non-linear dimensionality reduction (Supplementary Materials Figure S4). However, it is well known that various intrinsic and extrinsic factors will significantly affect the FTIR and density results. For the results to be transferable to archaeological and forensic contexts, the effect of the biological profile, the skeletal element and post-depositional bone diagenesis first need to be evaluated.

## Figures and Tables

**Table 2:** Measured/calculated FTIR indices and average CT density (CT).

	Temperature (°C)				
***Index***	**0**	300	600	900	1200
H1640	**1**	0.16 ± 0.096	0.035 ± 0.014	0.019 ± 0.004	0.012 ± 0.005
CN	**0.136**	0.034 ± 0.044	0.043 ± 0.028	0.163 ± 0.118	0.061 ± 0.078
H1415	**0.70**	0.19 ± 0.07	0.098 ± 0.021	0.028 ± 0.007	0.009 ± 0.003
H872	**0.0052**	0.0029 ± 0.0003	0.0027 ± 0.0005	0.0008 ± 0.0003	0.00006 ± 0.00001
H878	**0.0002**	0.0025 ± 0.0007	0.0023 ± 0.0005	0.0020 ± 0.0009	0.0010 ± 0.0015
SF	**3.17**	3.55 ± 0.17	4.29 ± 0.36	5.40 ± 0.35	4.81 ± 0.45
H960	**0.0003**	0.0016 ± 0.0001	0.0033 ± 0.0012	0.0122 ± 0.0032	0.0108 ± 0.0020
OH	**0.022**	0.078 ± 0.027	0.115 ± 0.022	0.183 ± 0.086	0.248 ± 0.080
CT	2139.8	1934.3 ± 195.1	1711.5 ± 89.1	2656.6 ± 294.8	3344.9 ± 216.5

## Data Availability

The datasets generated and/or analysed during the current study are available from the corresponding author on reasonable request.

## Supplementary Materials

The following supporting information can be downloaded at https://www.mdpi.com/article/10.3390/ma18040742/s1, Figure S1: Process of spectra manipulation; Figure S2: Peak measurement; Figure S3: Non-linear dimensionality reduction using Manifold Learning, a technique which finds a non-linear manifold within the higher-dimensional space and outputs new coordinates which correspond to a two-dimensional space (Orange Data Mining). Data is visualized with Scatter Plot; Table S1: Obtained indices.
