# Supplementary material for: Integrating Attenuated Total Reflectance–Fourier Transform Infrared Spectroscopy and Multidetector Computed Tomography for Analysis of Heat-Induced Changes in Bone
Source: Materials (Basel). 2025 Feb 7;18(4):742. doi: 10.3390/ma18040742 (PMC11857665; doi:10.3390/ma18040742)
Supplement: Supplementary file 1 [file materials-18-00742-s001.zip › materials-3371393-supplementary.pdf]

Integrating ATR-FTIR Spectroscopy and Multidetector CT for Analysis of Heat-Induced Changes in Bone

Tamara Leskovar [1], Fabio Cavalli [2], Lea Legan [3,4], Dario Innocenti [2], Polonca Ropret [3,5,6] and Matija Črešnar [1]

[1] University of Ljubljana, Faculty of Arts, Department of Archaeology, Centre for Interdisciplinary Research in Archaeology, Zavetiška 5, 1000 Ljubljana, Slovenia; *corresponding author: matija.cresnar@ff.uni-lj.si
[2] Research Unit of Paleoradiology and Allied Sciences - LTS - SCIT - Azienda Sanitaria Universitaria Giuliana Isontina, Via della pietà, 2/1 34100 Trieste, Italy;
[3] Institute for the Protection of Cultural Heritage of Slovenia, Poljanska cesta 40, 1000 Ljubljana, Slovenia;
[4] National Museum of Slovenia, Prešernova cesta 20, 1000 Ljubljana, Slovenia
[5] Museum Conservation Institute, Smithsonian Institution, 4210 Silver Hill road, 20746 Suitland, MD, USA
[6] University of Ljubljana, Faculty of Chemistry and Chemical Technology, Večna pot 113, 1000, Ljubljana, Slovenia

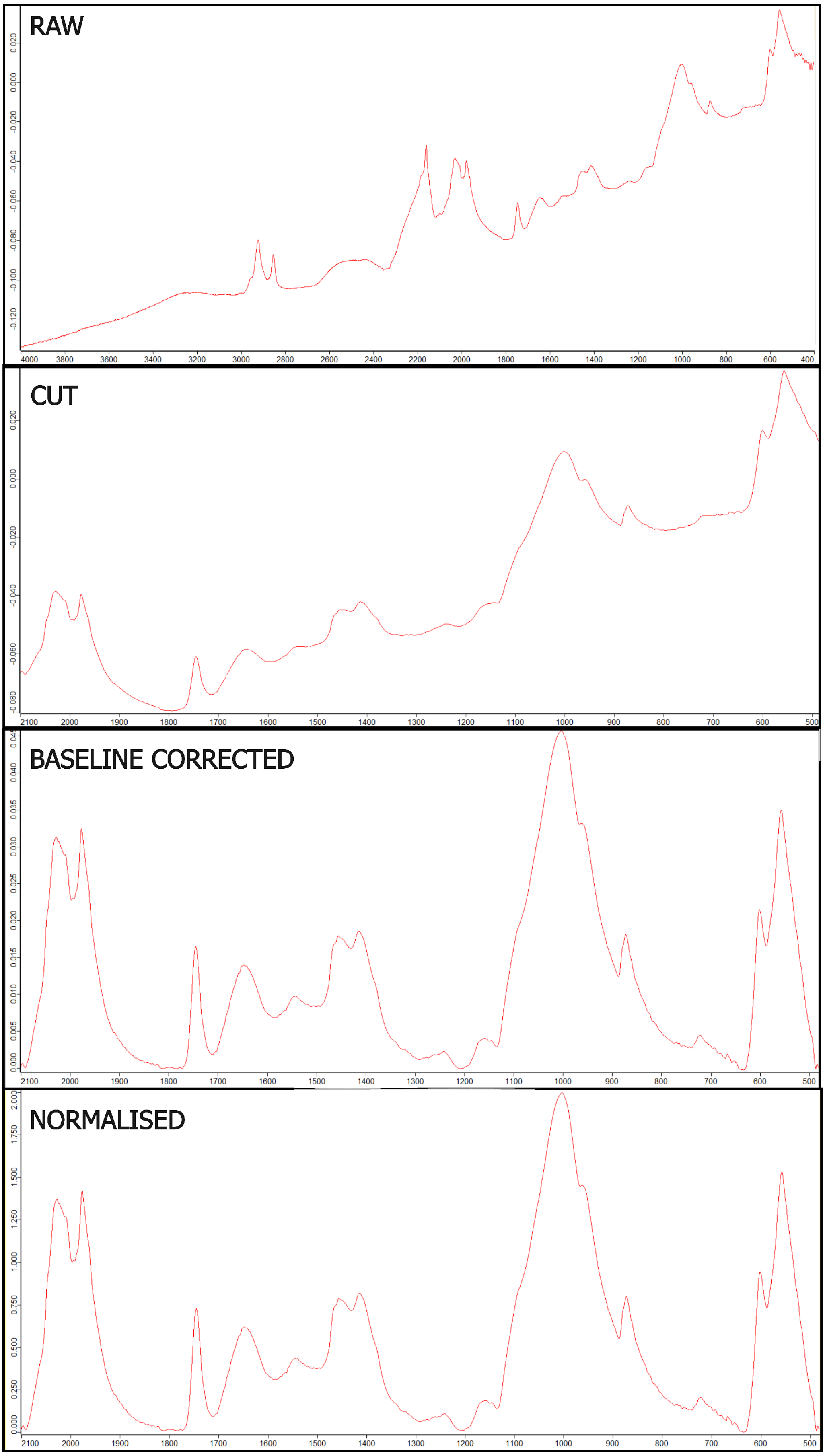

Figure S1. Process of spectra manipulation.

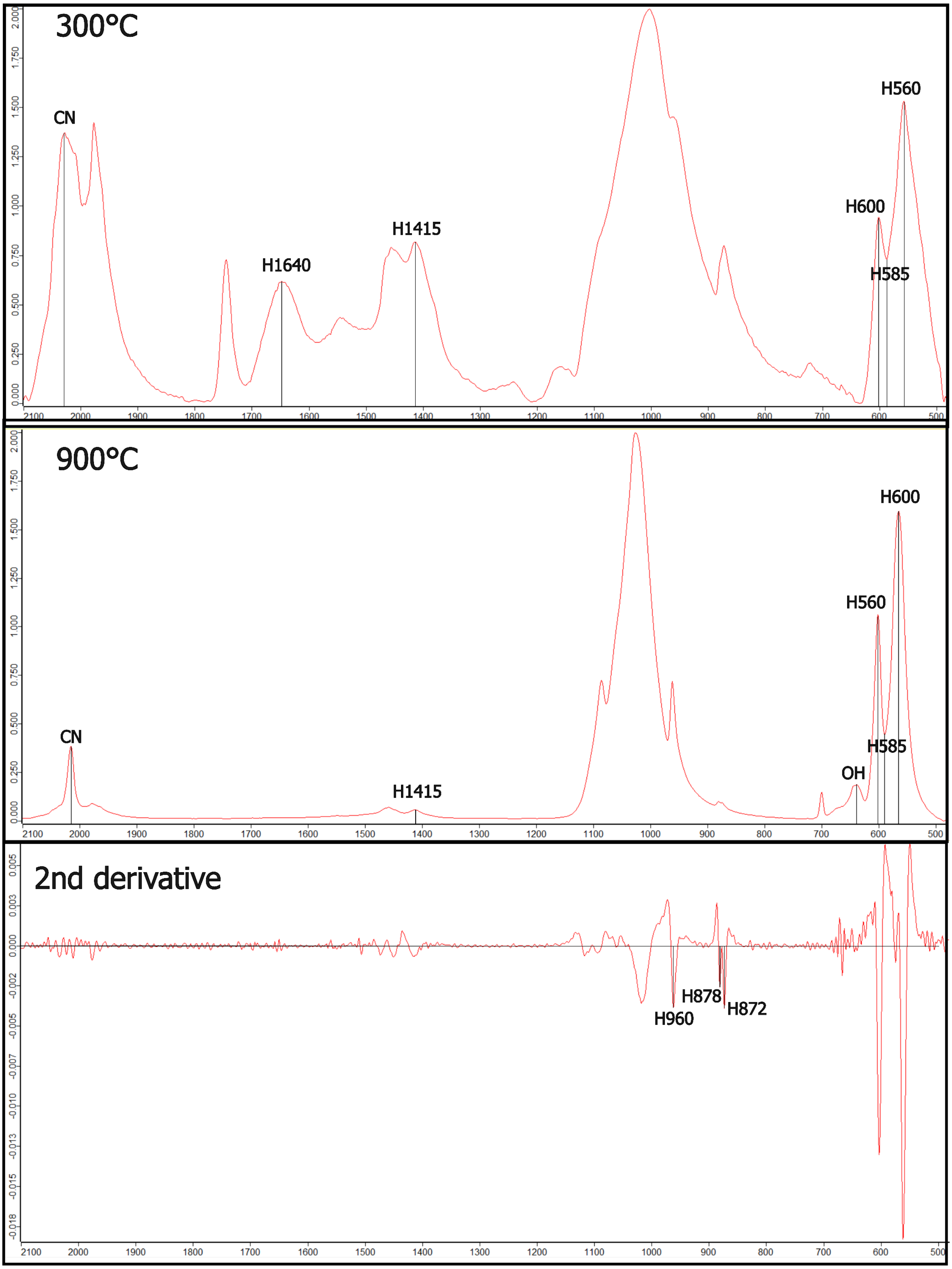

Figure S2. Peak measurement.

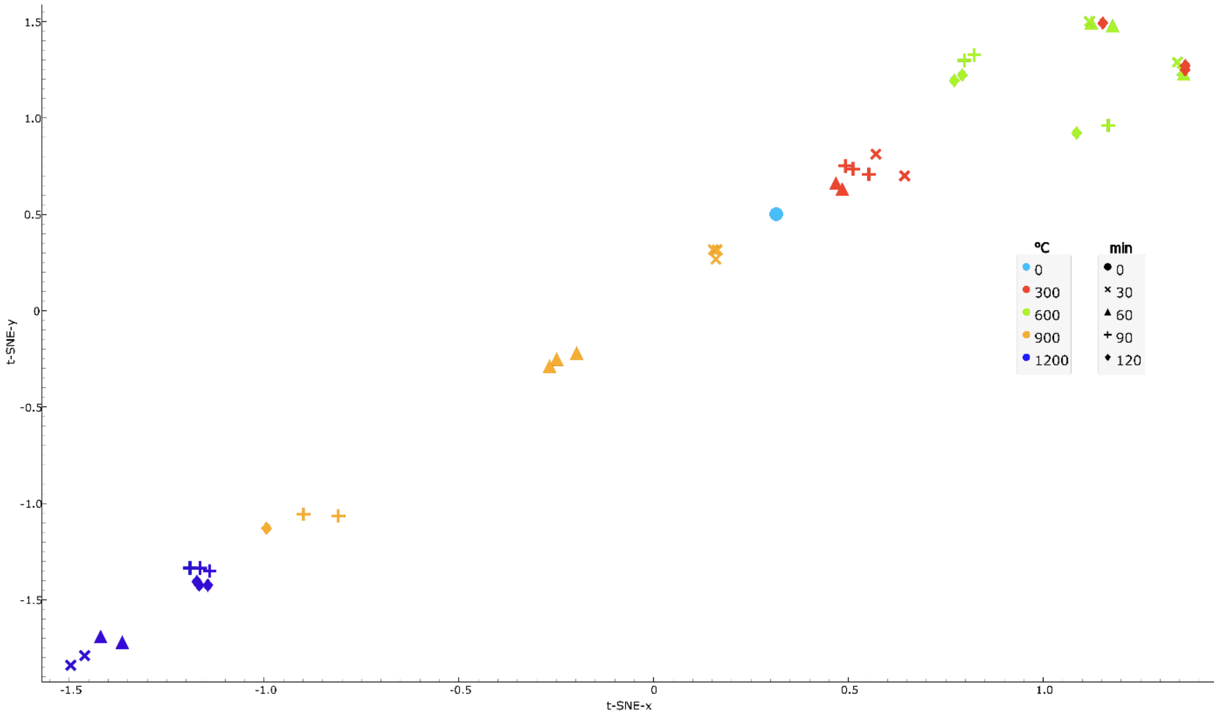

Figure S3. Non-linear dimensionality reduction using Manifold Learning, a technique which finds a non-linear manifold within the higher-dimensional space and outputs new coordinates which correspond to a two-dimensional space (Orange Data Mining). Data is visualized with Scatter Plot.

Table S1. Obtained indices.

SAMPLE	TEMPERATURE	TIME	SF	H1415	H1640	H872	H878	BPI	OH	CN/P	H960	CT
B0	0	0	3.168043	0.704296	1	0.005264	0.000195	5.663652	0.021683	0.016954	0.000325	2139.8
B1	300	30	3.237621	0.340681	0.349008	0.003497	0.003878	0.657464	0.031701	0.004766	0.001376	2023
B2	300	30	3.262978	0.335106	0.362564	0.003631	0.003871	0.618874	0.043472	0.169787	0.001582	2023
B3	300	30	3.9443	0.214205	1	0.001758	0.001653	0.824127	0.007841	0.050013	0.000761	2023
B4	300	60	3.438859	0.187758	0.170717	0.003077	0.002374	0.352668	0.082172	0.020051	0.001644	2078.7
B5	300	60	3.548427	0.1519	0.138939	0.002705	0.001879	0.266608	0.109291	0.029593	0.001523	2078.7
B6	300	60	3.565761	0.138747	0.130442	0.002671	0.001885	0.247683	0.113253	0.025287	0.001445	2078.7
B7	300	90	3.727486	0.139249	0.093026	0.002583	0.002263	0.298905	0.066005	0.00962	0.001513	2047.7
B8	300	90	3.595482	0.175456	0.170368	0.002608	0.001847	0.353773	0.07117	0.022849	0.001471	2047.7
B9	300	90	3.557575	0.171528	0.113602	0.002891	0.002487	0.327795	0.076528	0.023011	0.001569	2047.7
B10	300	120	3.801812	0.143796	0.091031	0.00267	0.002164	0.336093	0.062582	0.013249	0.001723	1617.2
B11	300	120	3.542485	0.157656	0.098455	0.002991	0.002499	0.291007	0.124726	0.034709	0.001698	1617.2
B12	300	120	3.738781	0.147987	0.072881	0.002807	0.002161	0.336667	0.077422	0.016269	0.00159	1617.2
B13	600	30	4.018983	0.116965	0.036513	0.003219	0.002791	0.26918	0.087037	0.015921	0.002464	1621.3
B14	600	30	4.019446	0.106325	0.050322	0.002806	0.002249	0.214829	0.116513	0.042035	0.002303	1621.3
B15	600	30	3.893515	0.127819	0.062448	0.003128	0.002756	0.24302	0.117839	0.055692	0.002171	1621.3
B16	600	60	4.330514	0.0949	0.026024	0.002642	0.002262	0.244915	0.081221	0.013456	0.002447	1625.5
B17	600	60	4.00086	0.119422	0.041224	0.003114	0.002743	0.258882	0.092909	0.019817	0.002299	1625.5
B18	600	60	4.075669	0.117106	0.041902	0.003153	0.002677	0.257373	0.094788	0.017886	0.00266	1625.5
B19	600	90	4.49922	0.082012	0.023213	0.002342	0.001826	0.182596	0.118118	0.012175	0.003744	1780
B20	600	90	4.020576	0.100541	0.048002	0.003167	0.002496	0.188257	0.137174	0.036107	0.002386	1780
B21	600	90	4.920571	0.060143	0.01706	0.001443	0.001224	0.14073	0.141198	0.016247	0.004536	1780
B22	600	120	5.044855	0.062447	0.016118	0.001833	0.00153	0.169872	0.10442	0.006015	0.005287	1819
B23	600	120	4.233936	0.104894	0.024883	0.002822	0.002313	0.212538	0.141756	0.003752	0.005231	1819
B24	600	120	4.377163	0.08298	0.027278	0.002593	0.002112	0.156609	0.145799	0.008448	0.004263	1819
B25	900	30	4.826798	0.039274	0.024109	0.001236	0.001148	0.076958	0.073979	0.131359	0.00622	2248.3
B26	900	30	5.607714	0.040722	0.009539	0.001397	0.00101	0.088837	0.062955	0.127839	0.007882	2248.3
B27	900	30	5.135097	0.037134	0.015794	0.001234	0.001092	0.076434	0.071265	0.120288	0.00716	2248.3

B28	900	60	5.722907	0.020564	0.008298	0.000489	0.00093	0.034045	0.150564	0.158133	0.014456	2506
B29	900	60	6.119441	0.022734	0.010233	0.000642	0.000966	0.044889	0.093897	0.162104	0.013498	2506
B30	900	60	5.037689	0.029426	0.010829	0.000981	0.001424	0.043977	0.181007	0.200502	0.013824	2506
B31	900	90	5.330969	0.024565	0.010442	0.000603	0.002739	0.043603	0.203134	0.064655	0.011073	2903.8
B32	900	90	5.350566	0.022971	0.012646	0.00049	0.002619	0.03706	0.288329	0.024793	0.013884	2903.8
B33	900	90	5.217244	0.028472	0.010278	0.000985	0.003349	0.048485	0.271074	0.020597	0.015063	2903.8
B34	900	120	5.829038	0.018136	0.008323	0.000318	0.002268	0.034674	0.238736	0.013417	0.014817	2968.2
B35	900	120	5.217244	0.028472	0.010278	0.000985	0.003349	0.048485	0.271074	0.020597	0.015063	2968.2
B36	900	120	5.350566	0.022971	0.012646	0.00049	0.002619	0.03706	0.288329	0.024793	0.013884	2968.2
B37	1200	30	4.953531	0.017531	0.023335	0.000133	0.000747	0.022906	0.092244	0.212628	0.009227	3625
B38	1200	30	4.841638	0.010484	0.010594	2.1E-05	0.002554	0.017977	0.233822	0.011463	0.009359	3625
B39	1200	30	4.47344	0.009544	0.014497	-9.4E-05	0.005318	0.014125	0.251795	0.050784	0.010814	3625
B40	1200	60	4.628704	0.00753	0.010074	7.75E-05	0.00095	0.010906	0.198667	0.046215	0.009396	3485.4
B41	1200	60	4.141696	0.00593	0.008356	6.26E-05	0.000168	0.008897	0.260581	0.009579	0.011643	3485.4
B42	1200	60	5.485013	0.007361	0.009964	8.34E-05	0.000301	0.010433	0.30073	0.017429	0.013163	3485.4
B43	1200	90	4.753932	0.010693	0.009435	8.99E-05	0.000917	0.0156	0.095735	0.115693	0.008812	3116
B44	1200	90	4.675133	0.011646	0.013826	9.63E-05	0.000231	0.016446	0.326338	0.011838	0.010039	3116
B45	1200	90	4.053218	0.009422	0.015503	0.00015	0.000128	0.012776	0.270418	0.018658	0.007798	3116
B46	1200	120	5.149033	0.007661	0.010586	4.97E-05	0.000209	0.010298	0.346409	0.016431	0.012696	3153
B47	1200	120	5.608593	0.0051	0.004242	2.89E-05	0.000164	0.00896	0.262812	0.004228	0.015053	3153
B48	1200	120	4.936341	0.008997	0.012341	0.000123	0.000127	0.012842	0.335488	0.010801	0.011635	3153
